# Structure of CTLA-4 complexed with a pH-sensitive cancer immunotherapeutic antibody

**DOI:** 10.1038/s41421-020-00202-9

**Published:** 2020-11-03

**Authors:** Han Gao, Haiyan Cai, Jia Liu, Xiaoxiao Wang, Pan Zheng, Martin Devenport, Ting Xu, Fei Dou, Yang Liu, Aiwu Zhou

**Affiliations:** 1 State Key Laboratory of Cognitive Neuroscience and Learning, and Beijing Key Laboratory of Genetic Engineering Drugs and Biotechnology, College of Life Sciences, Beijing Normal University, Beijing, 100875 China; 2 Key Laboratory of Cell Differentiation and Apoptosis of Chinese Ministry of Education, Department of Pathophysiology, Shanghai Jiao Tong University School of Medicine, Shanghai, 200025 China; 3 School of Life Science and Technology, Southeast University, Nanjing, Jiangsu 210096 China; 4 Alphamab Co. Ltd, Suzhou, Jiangsu 215125 China; 5 OncoImmune, Inc., Rockville, MD 20850 USA

**Keywords:** X-ray crystallography, Tumour immunology

Dear Editor,

Antibodies that target the immune system rather than the cancer cells have had a transformative impact for cancer therapy^1^. Ipilimumab, an antibody targeting cytotoxic T lymphocyte-associated antigen 4 (CTLA-4)^2^, was the first to receive regulatory approval for clinical use and thus played a major role in this breakthrough^3^. CTLA-4 has higher binding affinity to B7 ligands than CD28. CD28 is expressed on both naive and activated but not exhausted T cells and is required for T-cell activation. CTLA-4 is expressed after T-cell activation and was considered a negative regulator of T-cell function, although more recent studies demonstrated that CTLA-4 controls immune tolerance by enabling regulatory T (T_reg_) cells to rip B7 off the antigen-presenting cells, a process termed transendocytosis^4^. Anti-CTLA-4 antibodies have been shown to enhance T-cell proliferation and cytokine production in vitro^5,6^. However, numerous recent studies have shown that specific depletion of T_reg_ cells within the tumor microenvironment is associated with anti-CTLA-4-induced tumor rejection^7,8^. Ipilimumab, a fully human anti-CTLA-4 monoclonal antibody produced by Bristol Myers Squibb, could significantly increase overall survival in patients with advanced melanoma^3^. Several other CTLA-4 antibodies such as tremelimumab are also under development. However, the clinical usage of these CTLA-4 antibodies is often associated with severe immunotherapy-related adverse events (irAEs)^9^. These irAEs of CTLA-4 antibodies not only cause human suffering but also limit their doses and duration in cancer therapy, resulting in a suboptimal therapeutic outcome.

Recently, some of us reported a new generation of anti-CTLA-4 mAb that cause much less irAEs but showed even higher efficacy in inducing rejection of large established tumors and T_reg_ depletion in tumor tissues than both ipilimumab and treme-IgG1^10–12^. It is therefore of interest to delineate how the new anti-CTLA-4 antibodies differ from ipilimumab and tremelimumab in interacting with their shared target, CTLA-4. Here we prepared recombinant dimeric human CTLA-4 complexed with the Fab fragment of HL32 (HL32-Fab), a low-irAEs antibody developed recently^12^ (Supplementary Fig. S1), and solved the crystal structure of their complex at 3.05 Å resolution (Supplementary Table S1). The overall structure showed a tetramer containing a CTLA-4 homodimer and two copies of the HL32-Fab molecule (Fig. 1a). The CTLA-4 homodimer is like its apo form (PDB: 3OSK) with a root mean squared deviation of 2.9 Å, indicating the binding of HL32-Fab induced only minor conformational changes in CTLA-4. HL32-Fab mainly binds strands A and G of the front β-sheet and the FG loop of CTLA-4 through its heavy chain (V_H_) with its heavy chain complementarity determining region 3 (HCDR3). This region forms a long extended β-hairpin and anneals to the side of the front β-sheet of CTLA-4 (Fig. 1b). The binding interface involves extensive hydrophobic interactions formed by a cluster of aromatic residues such as Tyr104 and Tyr105 of CTLA-4, and Tyr102, Tyr107, Tyr109, and Tyr110 from V_H_ of the HL32-Fab (Supplementary Fig. S2a). There are only six hydrogen bonds in the interface, of which Asn106, Tyr107, and Tyr110 in the HCDR3 loop of HL32 form polar interactions with the main chain of residue Met3 on strand A, Leu106 on strand G, and Tyr104 on the FG loop in CTLA-4, respectively (Supplementary Fig. S2b and Table S2).

**Fig. 1:**
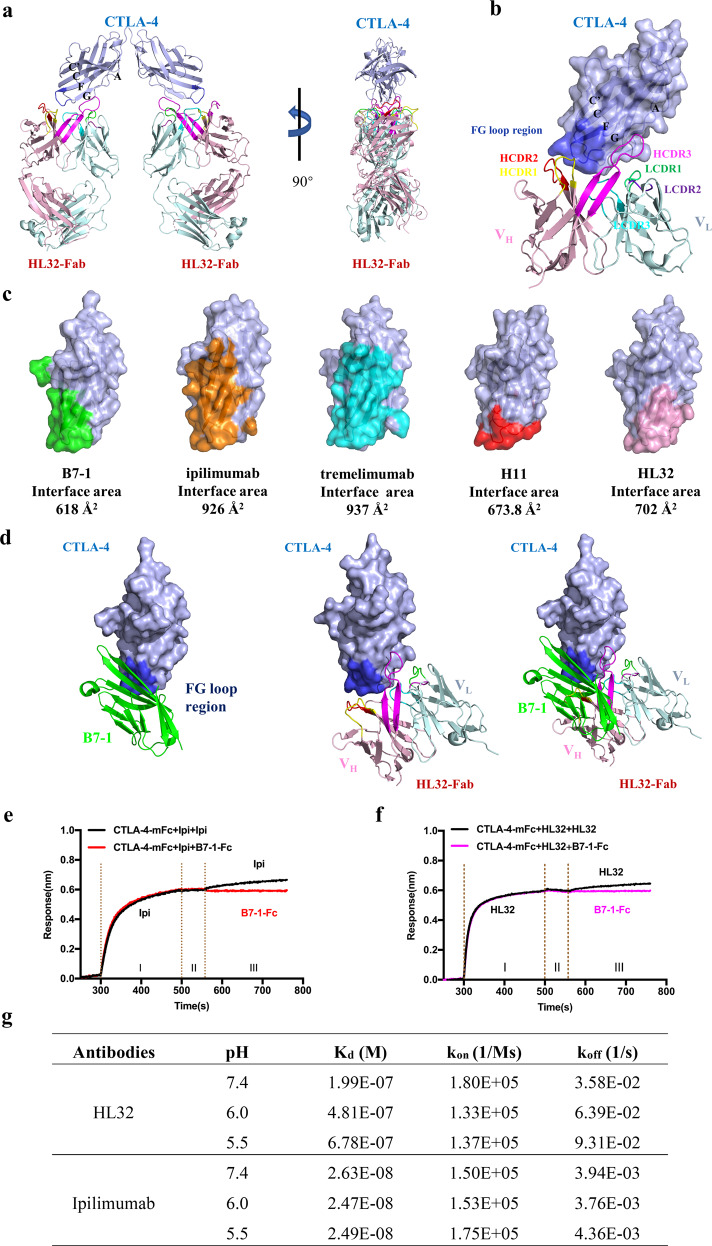
Crystal structure of the CTLA-4/HL32-Fab complex. **a** Overall structure of the HL32-Fab/CTLA-4 complex. There are a CTLA-4 homodimer and two HL32-Fab molecules in the complex with all four molecules aligned on a same plane. **b** Close-up view of the binding interface of HL32 and CTLA-4. CTLA-4 binds to the right corner of the front β-sheet of CTLA-4 with CTLA-4 shown as a semi-transparent surface (light blue). The variant heavy chain (V_H_, pink) and light chain (V_L_, pale-cyan) of HL32-Fab are shown as cartoon. The CDR1, CDR2, and CDR3 loops of V_H_ are colored in yellow, red, and magenta, respectively. The CDR1, CDR2, and CDR3 loops of V_L_ are colored in green, purple, and cyan, respectively. The MYPPPY motif of CTLA-4 FG loop is colored in blue. **c** CTLA-4 surface areas covered by B7-1 and various antibodies. The binding areas of B7-1 (PDB: 1I8L), ipilimumab (PDB: 5TRU), tremelimumab (PDB: 5GGV), H11 (PDB: 5E5M), and HL32 (PDB: 6XY2) on the CTLA-4 surface were colored in green, orange, cyan, red, and pink, respectively. The total buried surface area of CTLA-4 in the interface was calculated by PISA. **d** Superposition of the structures of CTLA-4/B7-1 (PDB: 1I8L) and CTLA-4/HL32-Fab shows potential clashes between B7-1 and HL32. **e**, **f** Binding analysis of B7-1 on preformed CTLA-4/antibody complexes. CTLA-4 was first immobilized on a sensor chip and subsequently loaded with ipilimumab or HL32 (stage I). After a brief wash (stage II), the chip was then loaded with B7-1-Fc (stage III) with the sensorgram followed. **g** Binding characteristics of HL32 and ipilimumab towards CTLA-4 monomer in different pH buffers. Antibodies were anchored on a protein A sensor and monomeric CTLA-4 solutions were flowed over the chip surface with sensorgrams recorded and binding *k*_on_ and *k*_off_ rates fitted.

The binding surface analysis showed that B7-1 binds to the lower half of the front β-sheet of CTLA-4 with a buried surface area of 618 Å^2^ (Fig. 1c). Ipilimumab and tremelimumab similarly bind across the front β-sheet of CTLA-4 and their binding surface areas, around 930 Å^2^, significantly overlap with the B7-1-binding position. Interestingly, the Fab of HL32 binds the right-bottom corner of the front β-sheet of CTLA-4 with a buried surface only about 700 Å^2^, which is close to that of the nanobody H11 (674 Å^2^), but significantly smaller than those of the ipilimumab and tremelimumab Fab fragments. The binding topology of HL32 differs to that of ipilimumab or tremelimumab where the HL32-Fab/CTLA-4 complex is “taller” than those of ipilimumab or tremelimumab (Supplementary Fig. S3). Furthermore, the binding area of B7-1 on CTLA-4 involves Arg35, Lys95, and Glu97 from the hydrophilic patch and part of the hydrophobic area (Supplementary Fig. S4). The binding area of ipilimumab or tremelimumab is extensive and covers a large area including both patches. In contrast, HL32 binding involves mainly the hydrophobic area. This emphasizes that hydrophobic interactions play the dominant role in stabilizing the binding between HL32 and CTLA-4.

Protein interface surface analysis shows that residues from the lower part of strands F and G of the CTLA-4 front β-sheet and their connecting loop are largely involved in the complex formation (Supplementary Fig. S5). Notably, the ^97^MYPPPY^102^ motif in the FG connecting loop of CTLA-4, essential for interaction with the B7 ligands, is involved in the binding of all the antibodies, including HL32. This indicates that HL32 and B7-1 have an overlapped binding surface on CTLA-4 (Fig. 1d). More specifically, there would be steric clashes between Leu97, Glu99 of B7-1, and Tyr102, Tyr107 in the HCDR3 loop of HL32 if HL32 and B7-1 were to form ternary complex with CTLA-4 (Supplementary Fig. S6, colored orange). Thus, HL32 and B7-1 could not bind CTLA-4 simultaneously. To confirm this, we then evaluated the binding properties of HL32 by Bio-Layer Interferometry (BLI) assay, where CTLA-4 was first immobilized on a sensor chip and subsequently loaded with HL32 or control antibody ipilimumab (stage I). After a brief wash (stage II), the chip was then loaded with B7-1-Fc (stage III). If B7-1 would bind to the preformed CTLA-4/antibody complex on the chip, one would observe an increased signal on the sensorgram. As expected, there was no increased signal when B7-1-Fc was loaded onto the sensor coated with preformed CTLA-4/ipilimumab complex (Fig. 1e, red curve, stage III). Likewise, no signal increase was observed from the chip loaded with preformed CTLA-4/HL32 complex either (Fig. 1f, magenta curve, stage III) even though the binding avidity of HL32 towards CTLA-4 is about threefold lower than that of ipilimumab^12^. This clearly indicates that a stable binding of these antibodies on the cell surface CTLA-4 would likely prevent CTLA-4 from binding to B7 on the opposing cells if the latter is presented subsequently to HL32–CTLA-4 interaction. In our previous studies^12^, it was noted that HL32 cannot compete with B7-1 for the binding to CTLA-4 when both were added at the same time; thus, it was considered non-blocking under physiological condition. Based on our current structural studies, HL32 and ipilimumab bind to epitopes that overlap with the B7-1-binding site. Indeed, BLI assay suggests both may be called blocking antibodies. However, we would like to emphasize that competitive binding differs from the BLI assay as the relative affinity and more specifically, on-rate of B7-1 vs. HL32 to CTLA-4 would have more effect on the overall outcome^12^. Likewise, ipilimumab has been shown to be ineffective in competing with cell surface B7-1 in their binding to CTLA-4^12^. Therefore, mutual exclusivity in binding site does not necessarily guarantee a physiological blocking of the B7–CTLA-4 interaction.

As HL32 and ipilimumab do not differ fundamentally in their potential in preventing the binding of B7-1 to CTLA-4 once the antibody binds their target regardless of their relative binding affinities towards CTLA-4, their difference in safety and therapeutic effect should not be explained on the basis of differential blocking activities. An important advance in understanding the physiological function of CTLA-4 is that CTLA-4 undergoes constitutive recycling and mediates transendocytosis of B7. Genetic inactivation of this process leads to autoimmune diseases in human^13^. In this context, it is plausible that antibodies binding on CTLA-4 could have different effects on these processes, ultimately leading to different toxicity. Some of us demonstrated that ipilimumab and treme-IgG1 interfered with CTLA-4 recycling causing lysosomal degradation of CTLA-4, whereas HL32 preserves CTLA-4 recycling as it rapidly dissociates from CTLA-4 after endocytosis due to its sensitivity to late endosomal and lysosomal pH^11^. Here we further confirmed this pH-dependent dissociation of the CTLA-4/HL32 complex by measuring their binding affinity at different pHs using monomeric CTLA-4 protein (Fig. 1g). This shows that the affinity of HL32 was decreased about 3.4-fold from pH 7.4 to pH 5.5, whereas the affinity of ipilimumab remained largely unchanged (Fig. 1g). Detailed structural analysis showed that there are four histidine residues (His4 from CTLA-4, and His60, His90, and His101 from HL32-Fab) near the binding interface of CTLA-4 and HL32 (Supplementary Fig. S7). Changes of pH could affect the charge state of imidazole rings of these histidine residues leading to altered local interactions, thereby perturb the binding interactions between HL32 and CTLA-4. As the crystals of HL32/CTLA-4 were grown at pH 5.6 and the sidechains of Lys30 of CTLA-4 and His60 in V_H_ of HL32 is only about 4 Å apart in the structure, it is highly probable that acidic pH disrupts the potential stabilizing interaction between these two residues resulting in dissociation of the complex (Supplementary Fig. S7).

Overall, this study provides novel information that the distinct different toxicity of CTLA-4 antibodies cannot be solely explained by their potential in blocking CTLA-4/B7 interaction. The lower toxicity of HL32 likely arises from its unique binding site on CTLA-4, which may have less detrimental effect on CTLA-4 interactions with non-B7 partners that enable recycling^11^. This sheds light on the development of novel CTLA-4 antibodies targeting different surface areas of CTLA-4 for safer cancer immunotherapy.

## Supplementary information

supplementary information

## Data Availability

Coordinates and structure factor of the structure reported here have been deposited into the Protein Data Bank with PDB code 6XY2.

## Supplementary information

**Supplementary Information** accompanies the paper at (10.1038/s41421-020-00202-9).
