# Supplementary material for: Structure of CTLA-4 complexed with a pH-sensitive cancer immunotherapeutic antibody
Source: Cell Discov. 2020 Nov 3;6:79. doi: 10.1038/s41421-020-00202-9 (PMC7606454; doi:10.1038/s41421-020-00202-9)
Supplement: Supplementary file 1 — supplementary information [file 41421_2020_202_MOESM1_ESM.pdf]

Supplementary information

Materials and Methods

Cell lines and culture

The 293F suspension cell lines were purchased from Thermo Fisher. For protein expression, 293F cells were cultured in Gibco 293F SFM II medium (Thermo Fisher) and kept at 37 degrees Celsius in an 8% CO_2 incubator.

Protein purification and complex preparation

The Fab fragment of HL32 was produced using the 293F mammalian expression system[1]. The cDNAs encoding the heavy chain (VH-CH1) with an 8×His tag and light chain (VL-CL) of HL32 were cloned into pCEP4 expression vectors. They were co-transfected into 293F suspension cells using Polyethylenimine (PEI). The secreted Fab fragment (HL32-Fab) in the medium was purified by a Ni-NTA column (Roche) following standard protocol. Recombinant glycosylated homodimeric (residues 3-125, including Cys122 responsible for the interchain disulfide bond) form of human CTLA-4 IgV domain (amino acids 3-125) with a C-terminal His-tag, was expressed in 293F cells using a similar procedure as HL32-Fab. The secreted protein in the medium was first purified by a Ni-NTA column and the eluted samples were then mixed with the purified HL32-Fab. The mixture was further purified by ion-exchange column chromatography followed by gel filtration chromatography (Hiload 26/600 Superdex 75pg). The protein qualities were evaluated by SDS-PAGE gel stained with Coomassie blue.

Crystallization of CTLA-4/HL32 complex

Purified CTLA-4 dimer and HL32-Fab complex was concentrated to ~20 mg/mL and the initial crystallization was performed with commercially available screening

solutions through the sitting-drop vapor diffusion method. After optimizing the initial crystallization conditions, crystals suitable for data collection were obtained at room temperature from 0.8M lithium sulfate, 0.5M ammonium sulfate and 0.1M sodium citrate pH 5.6.

Structure determination and refinement

Crystals were cryo-protected with 20% glycerol in the mother liquor and flash-cooled in liquid nitrogen. Diffraction data were indexed and processed with iMosflm and scaled using the Aimless software from the CCP4 suite[2]. The initial phases were obtained by molecular replacement using Phaser[3] with CTLA-4 (PDB 3OSK), Fab fragment model (PDB 5JDS), respectively. The models were subsequently manually rebuilt using Coot[4] and refined to good geometry using Refmac[5]. The atomic coordinates and structure factors have been deposited in the Protein Data Bank (PDB) (**Supplement information, Table S1**). The binding interface between CTLA-4 and HL32-Fab was analyzed by PISA[6]. Figures were created using PyMOL software[7].

Evaluation of the effect of antibodies on CTLA-4 and B7 binding

A BLI (bio-layer interferometry) assay (Octet K2) was used to assess the competitive binding activity of the anti-CTLA-4 antibody on CTLA-4-B7 binding. Human CTLA-4 fused mouse Fc (CTLA-4-mFc) protein was diluted to 10 μg/mL, anchored on the Anti-Mouse Fc Capture (AMC) biosensors for 150 seconds, loaded with HL32 (100 nM) for 200 seconds (stage I) and then washed with PBST for 60 seconds (stage II). Subsequently the biosensors were dipped into the same antibody solution or 400 nM B7-1-Fc for 200 seconds (stage III). The binding response sensorgram was followed.

The pH dependent binding antibody on CTLA-4

HL32 or ipilimumab antibodies was diluted to 10μg/ml, anchored on a protein A chip

biosensor for 150 seconds. Monomeric CTLA-4-His solutions (25, 50, 100, 200,400, 900nM for ipilimumab and 625, 1250nM, 2500nM, 5000nM and 10000nM for HL32) were flowed over the chip surface with sensorgrams recorded. The binding k_{on} and k_{off} rates were fitted with a 1:1 binding model.

References

1 L'Abbé, D., Bisson, L., Gervais, C., Grazzini, E. & Durocher, Y. Transient Gene Expression in Suspension HEK293-EBNA1 Cells: Methods and Protocols. 1-16 (2018).

2 Collaborative, C. P. The CCP4 suite: programs for protein crystallography. *Acta crystallographica. Section D, Biological crystallography* **50**, 760-763 (1994).

3 Storoni, L. C., McCoy, A. J. & Read, R. J. Likelihood-enhanced fast rotation functions. *Acta Crystallographica Section D: Biological Crystallography* **60**, 432-438 (2004).

4 Emsley, P. & Cowtan, K. Coot: model-building tools for molecular graphics. *Acta Crystallographica Section D: Biological Crystallography* **60**, 2126-2132 (2004).

5 Winn, M., Isupov, M. & Murshudov, G. N. Use of TLS parameters to model anisotropic displacements in macromolecular refinement. *Acta Crystallographica Section D: Biological Crystallography* **57**, 122-133 (2001).

6 Krissinel, E. & Henrick, K. Inference of macromolecular assemblies from crystalline state. *Journal of molecular biology* **372**, 774-797 (2007).

7 Delano, W. L. The PyMol Molecular Graphics System. *Proteins Structure Function & Bioinformatics* **30**, 442-454 (2002).

Supplementary Table S1. Crystallographic data collection and refinement statistics

	CTLA-4/HL32-Fab
PDB code	6XY2
Crystallization condition	0.8 M lithium sulfate, 0.5 M ammonium sulfate, 0.1 M Na citrate pH 5.6
**Data collection**	
space group	C 2 2 21
a, b, c (Å)	163.812, 254.131, 66.847
α, β, γ (°)	90, 90, 90
Wavelength (Å)	0.9789
Resolution (Å)	81.93-3.05 (3.18-3.05)
Total NO. of observation	273494 (41786)
Total NO. unique	28576 (4580)
R_{merge}	0.128 (1.385)
I/σI	8.5 (1.4)
Completeness (%)	99.9 (100)
Multiplicity	9.6 (9.1)
$CC_{1/2}$	0.996(0.686)
**Refinement**	
Resolution (Å)	75.33-3.05
No. of reflections	27133
No. of residues	553
No. of atoms	4163
R_{work}/R_{free}	0.2486/0.2925
B-factors (Å^2)	117.3
Bond length RMSD(Å)	0.002
Bond length RMSD(°)	0.553
**Ramachandran plot**	
In Preferred Region (%)	92.66
In Allowed Region (%)	7.16
Outliers (%)	0.18

Supplementary Table S2. Polar interactions between HL32-Fab and CTLA-4 (distance ≤ 3.7Å)

HL32-Fab contact residue (atom)	HL32-Fab residue location	CTLA-4 contact residue (atom)	CTLA-4 residue location
N106 (ND2)	HCDR3	M3 (O)	A strand
Y110 (OH)	HCDR3	Y104 (N)	FG loop
Y110 (OH)	HCDR3	Y104 (O)	FG loop
Y107 (OH)	HCDR3	L106(N)	G strand
Y107 (OH)	HCDR3	L106(O)	G strand
L91 (O)	LCDR1	Y105 (OH)	FG loop

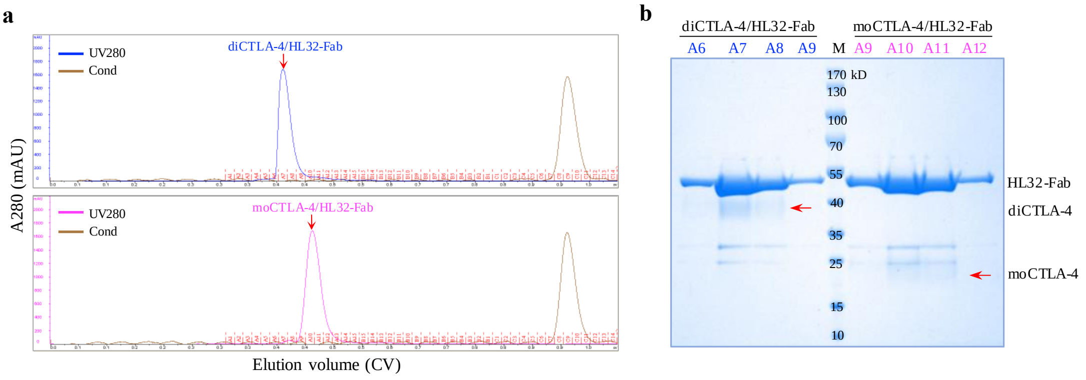

Supplementary Figure S1. Preparation of recombinant dimeric or monomeric human CTLA-4 complexed with HL32-Fab. CTLA-4 dimer is formed by an intermolecular disulfide bond of Cys122. **a** When mixed with HL32-Fab, the mixture was loaded on a Hiload 26/600 Superdex 75pg gel filtration column, single peak was obtained in elution, respectively. The gel filtration elution profile of dimeric human CTLA-4 complexed with HL32-Fab is shown in the upper panel, while the elution of monomeric human CTLA-4 complexed with HL32-Fab is shown in the lower panel. **b** The complexes in each peak was analyzed by SDS-PAGE. (diCTLA-4: CTLA-4 dimer; moCTLA-4: CTLA-4 monomer)

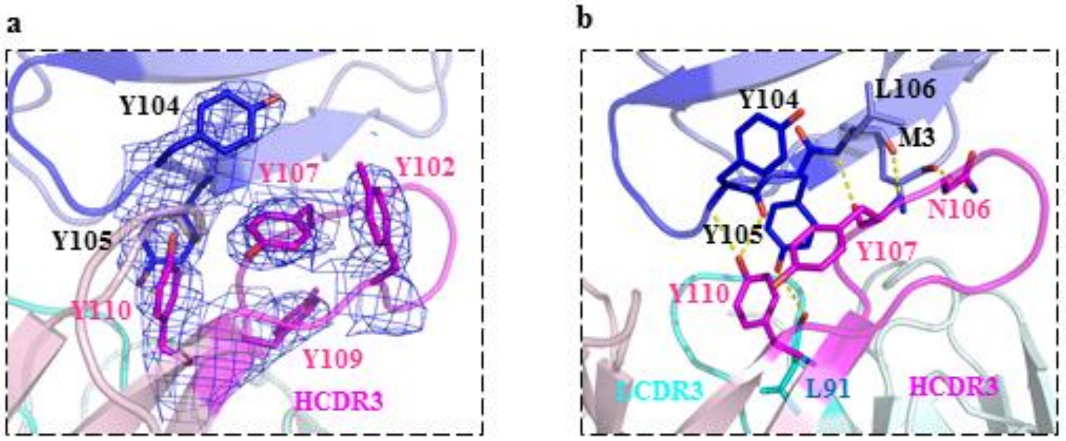

Supplementary Figure S2. The binding interface of the CTLA-4/HL32-Fab complex. a A cluster of Tyrosine residues from HCDR3 and CTLA-4 formed hydrophobic interactions. The 2Fo-Fc electron density map covering these residues are shown as blue mesh and contoured at 1 sigma. **b** There are six hydrogen bonds (dashed yellow lines) in the HL32-Fab/CTLA-4 binding interface.

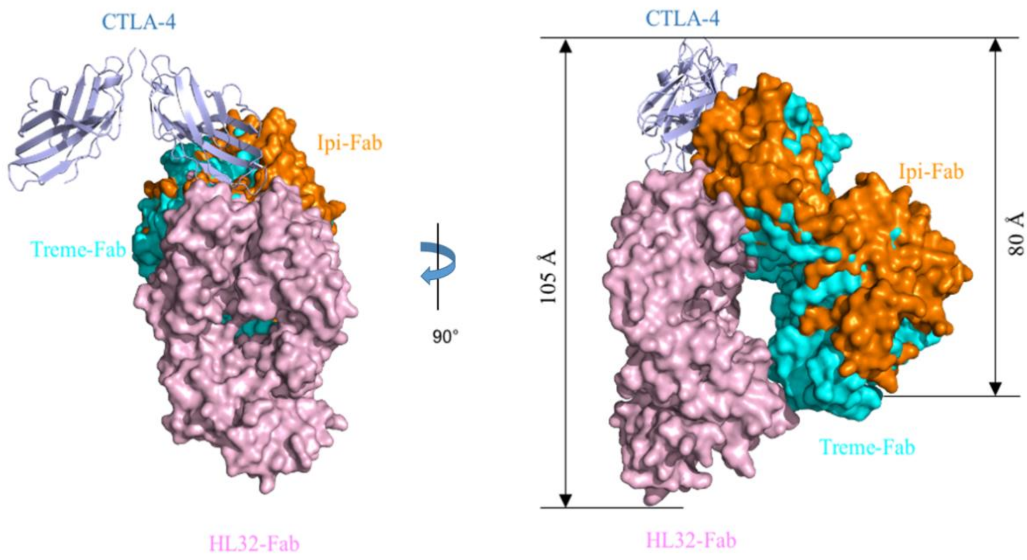

Supplementary Figure S3. **the overall topology of CTLA-4 complexed with the Fab of HL32 or ipilimumab or tremelimumab.** Superposition of the structures of CTLA-4/HL32-Fab (PDB: 6XY2)、CTLA-4/ipilimumab-Fab (PDB: 5TRU) and CTLA-4/tremelimumab-Fab (PDB: 5GGV) shows the binding orientation. The distance between the cell membrane, where the CTLA-4 dimer is anchored, and HL32-Fab is about 105Å, while that of ipilimumab-Fab or tremelimumab-Fab is about 80Å.

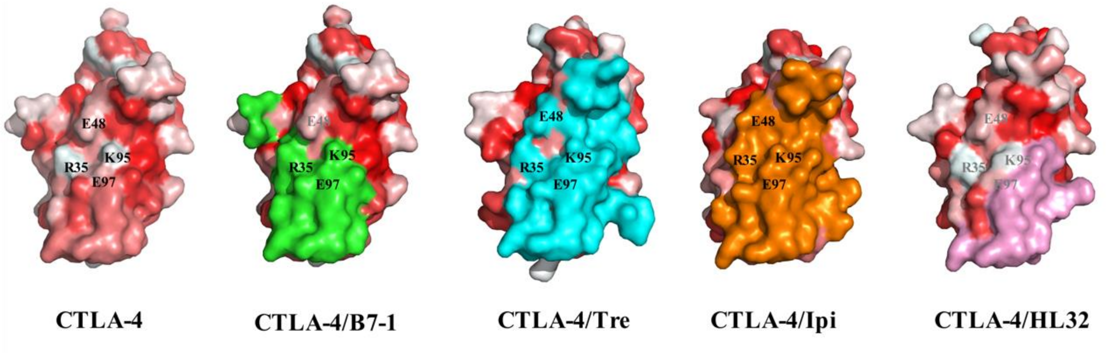

Supplementary Figure S4. CTLA-4 surface covered by B7-1 and various antibodies. The hydrophobicity analysis shows that the front CTLA-4 surface has a large hydrophobic patch (red) and a hydrophilic patch (white) involving fours residues (R35, E48, K95 and E97). B7-1 binding area on CTLA-4 (green) covers part of the hydrophobic patch and involves three of the four hydrophilic residues. The binding areas of ipilimumab (orange) and tremelimumab (cyan) span over the whole front beta-sheet including both hydrophobic and hydrophilic patches. In contrast, HL32 binding area (pink) only covers part of the hydrophobic area.

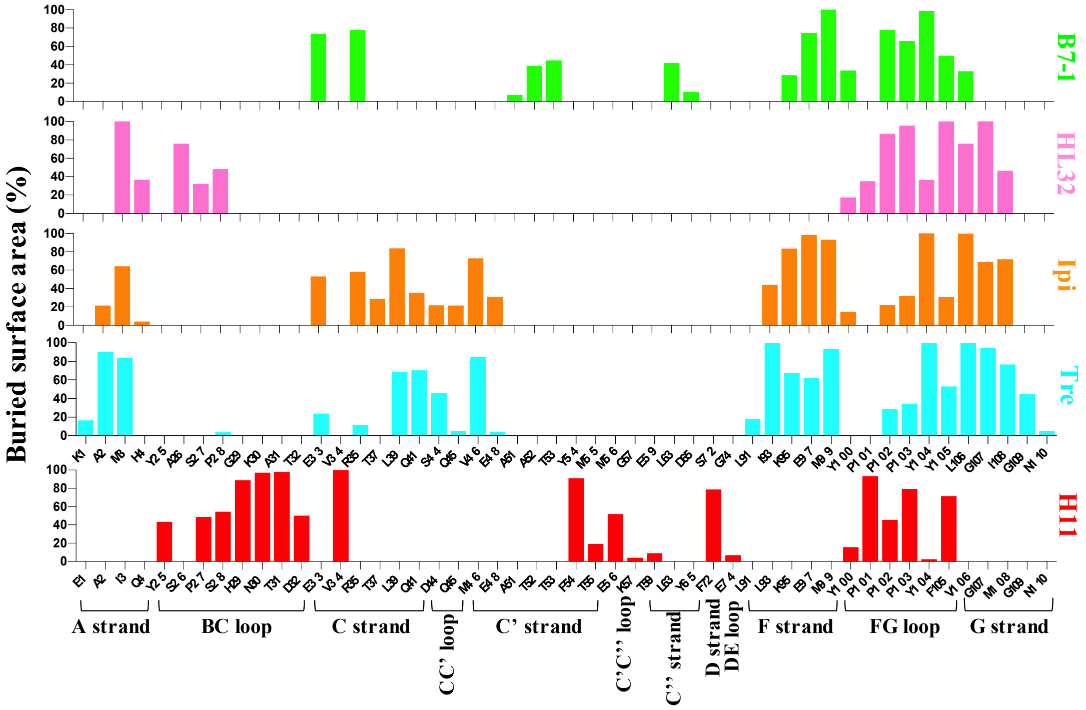

Supplementary Figure S5. The CTLA-4 residues involved in complex formation. The percentage of the buried surface area of each CTLA-4 residue involved in the interface was analyzed by PISA and plotted together. This indicates that the FG loop of CTLA-4 is involved in the formation of all the CTLA-4 complexes with B7-1, HL32, Ipilimumab, Tremelimumab and H11.

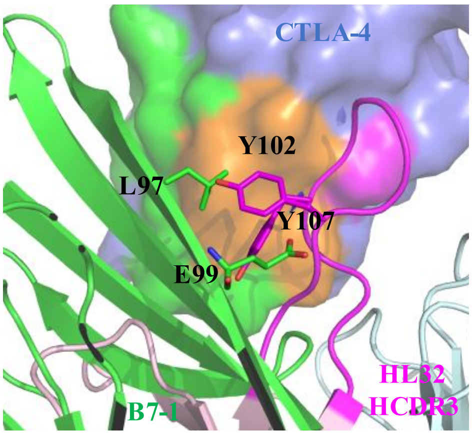

Supplementary Figure S6. B7-1 and HL32 have an overlapped binding area (orange) on CTLA-4. Residues Tyr102 and Tyr107 in HCDR3 loop of HL32 would clash with Leu97 and Glu99 of B7-1 if HL32 and B7-1 bind CTLA-4 simultaneously. CTLA-4 surface is shown in slate. B7-1, the V_H and V_L of HL32-Fab are shown green, pink and pale-cyan cartoon, respectively. Selected residues are shown in sticks.

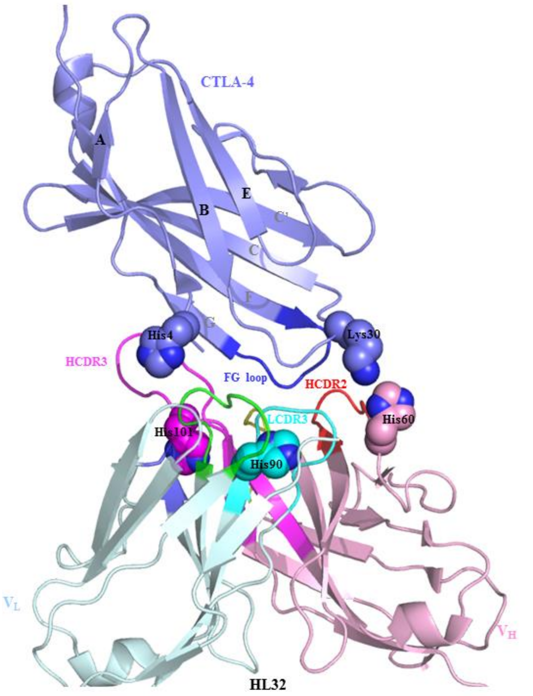

Supplementary Figure S7. **Histidine residues near the binding interface of CTLA-4 and HL32-Fab in the crystal structure of HL32-Fab/CTLA-4 complex**. His4 and Lys30 of CTLA-4, His60, His90 and His101of HL32 are shown in spheres. The sidechains of Lys30 and His60 are about 4Å apart in the structure where the crystals were grown at pH5.6, but they could form hydrogen bonding interactions under neutral pH. CTLA-4 (slate), the heavy chain (VH, pink) and light chain (LH, cyan) of HL32-Fab are shown as cartoon representations. The CDR1, CDR2 and CDR3 loops of VH are colored in yellow, red and magenta, respectively. The CDR1, CDR2, and CDR3 loops of VL are colored in green, purple and cyan, respectively. The FG loop of CTLA-4 is colored blue.
